# Natural Iminosugar (+)-Lentiginosine Inhibits ATPase and Chaperone Activity of Hsp90

**DOI:** 10.1371/journal.pone.0043316

**Published:** 2012-08-20

**Authors:** Fabrizio Dal Piaz, Antonio Vassallo, Maria Giovanna Chini, Franca M. Cordero, Francesca Cardona, Claudio Pisano, Giuseppe Bifulco, Nunziatina De Tommasi, Alberto Brandi

**Affiliations:** 1 Dipartimento di Scienze Farmaceutiche e Biomediche, Università di Salerno, Fisciano, Italy; 2 Dipartimento di Chimica, Università degli Studi della Basilicata, Potenza, Italy; 3 Departimento di Chimica “Ugo Schiff”, Università of Firenze, Sesto Fiorentino, Italy; 4 Sigma-Tau, Pomezia, Italy; Fred Hutchinson Cancer Research Center, United States of America

## Abstract

Heat shock protein 90 (Hsp90) is a significant target in the development of rational cancer therapy due to its role at the crossroads of multiple signaling pathways associated with cell proliferation and cell viability. The relevance of Hsp90 as a therapeutic target for numerous diseases states has prompted the identification and optimization of novel Hsp90 inhibitors as an emerging therapeutic strategy. We performed a screening aimed to identify novel Hsp90 inhibitors among several natural compounds and we focused on the iminosugar (+)-lentiginosine, a natural amyloglucosidases inhibitor, for its peculiar bioactivity profile. Characterization of Hsp90 inhibition was performed using a panel of chemical and biological approaches, including limited proteolysis, biochemical and cellular assays. Our result suggested that the middle domain of Hsp90, as opposed to its ATP-binding pocket, is a promising binding site for new classes of Hsp90 inhibitors with multi-target anti-cancer potential.

## Introduction

Heat shock protein 90 (Hsp90) is an abundant molecular chaperone involved in a variety of cellular processes ranging from signal transduction to viral replication. Hsp90 is involved in the folding, stabilization, activation and assembly of a wide range of “client” proteins, thus playing a central role in many cellular processes [1]. Since multiple oncogenic proteins are substrates for the Hsp90 mediated protein folding process, this molecular chaperone has emerged as an exciting target for the development of cancer chemotherapeutics. In addition, studies suggested that certain Hsp90 inhibitors accumulate in tumor cells more effectively than in normal tissue, leading to >200 fold differential selectivity [2]. Coupled with the observation that Hsp90 is over-expressed in a variety of human malignancies, the development of Hsp90 inhibitors has become an attractive chemotherapeutic approach [3]–[8]. To date, many clinical and translational researches on Hsp90 inhibitors have been based on gendanamycin derivatives, such as 17-allylamino-17-demethoxygeldanamycin (17-AAG) or 17-dimethylaminoethylamino-17-demethoxygeldanamycin (17-DMAG). Some difficulties encountered clinically with these inhibitors include hepatic toxicity and drug administration through intravenous infusion [9]. These issues highlight a critical need for novel and improved inhibitor. Medicinal plants and their active metabolites are one of the major sources of chemical diversity for starting materials in drug discovery for the treatment of human diseases including cancer [10], [11]. Such medicinal plants often contain small alkaloids as the active principles, which have the advantage of being orally available [12].

On this basis, we performed a screening oriented to obtain Hsp90 inhibitors on several natural compounds and we focused on the iminosugar (+)-lentiginosine for its chemical and bioactivity characteristics. (+)-Lentiginosine, a natural occurring dihydroxyindolizidine alkaloid, has been shown to be a selective and the most powerful indolizidine inhibitor of amyloglucosidases [13], [14]. Besides, recently the synthetic enantiomer (−)-lentiginosine was found a proapoptotic inducer with low cytotoxicity [15], [16].

Herein we report the study of Hsp90 inhibitory activity of (+)-lentiginosine and some its synthetic derivatives, performed by means of a panel of chemical and biological approaches. Our results demonstrated that *in vitro* (+)-lentiginosine represent an innovative scaffold for the design of new Hsp90 inhibitors interacting with the protein middle domain. Since glycosidase inhibitors have received growing attention as potential therapeutic agents for several pathologies, also including tumor metastasis [17]–[19], the anti-Hsp90 effects of (**+**)**-**lentiginosine, together with its anti-amyloglucosidases activity could indicate this compound as a very interesting lead for the design of a new class of multifunctional inhibitors.

## Results and Discussion

**Figure 1:**
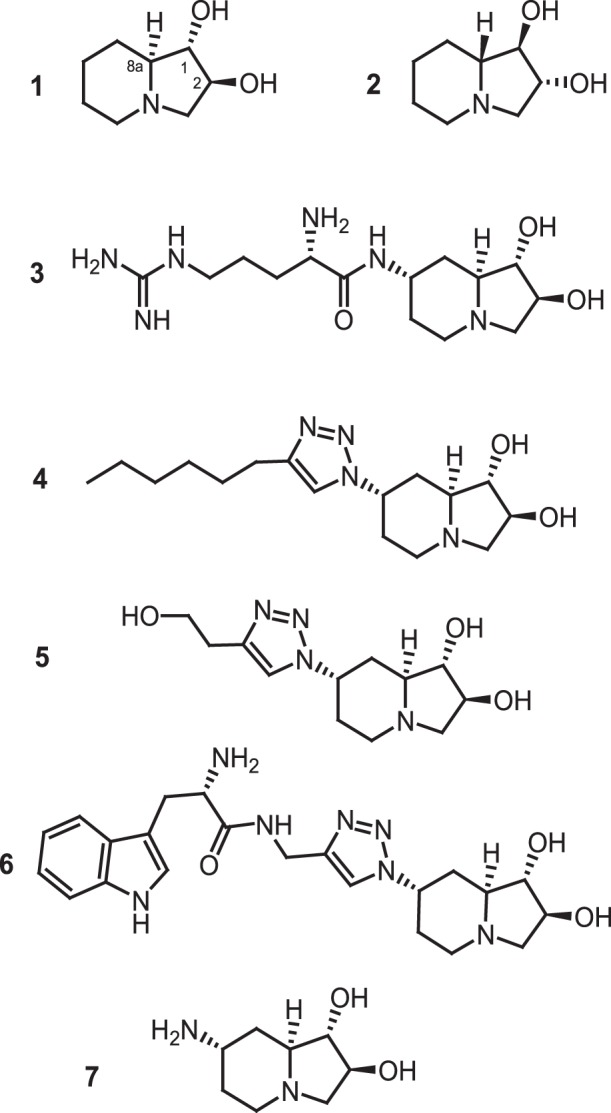
Structure of compounds 1–7.

In order to evaluate the ability of iminosugars to affect the biological activity of Hsp90, we started a chemical-biological study using (+)-lentiginosine (compound **1** in Figure 1) as model compound; it was selected because of its simple structure, high water solubility, and significant biological activity [13], [14].

**Figure 2:**
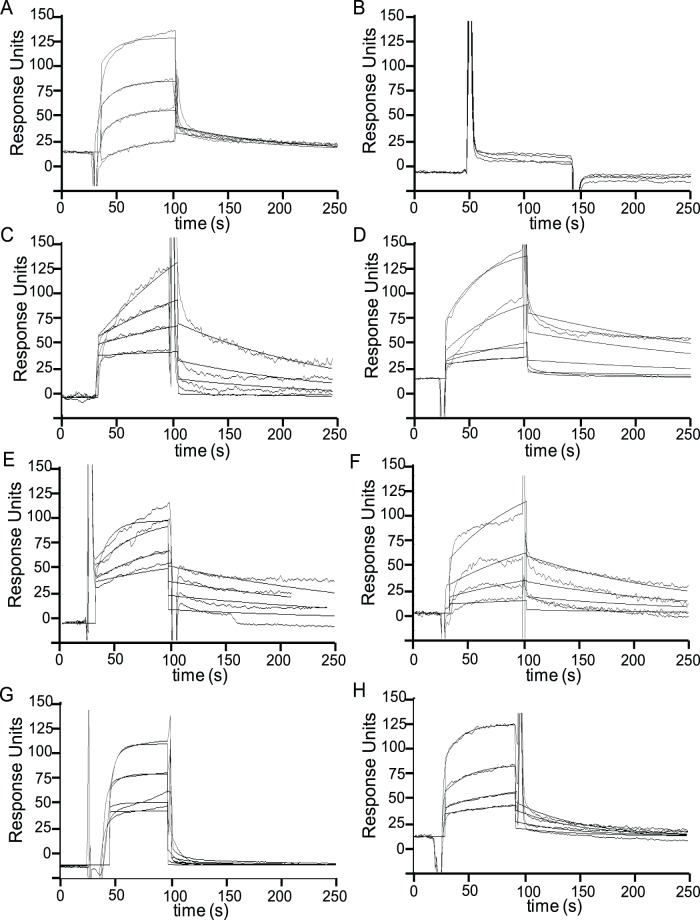
SPR analysis results. Sensorgrams obtained by injecting different concentrations (from 0.020 to 1 *µ*M) of **1** (A), **2** (B), **3** (C), **4** (D), **5** (E), **6** (F) **7** (G) and radiciol (H) on immobilized Hsp90.

As a first step, we evaluated of the ability of compound **1** to interact with Hsp90 by a surface plasmon resonance (SPR) based binding assay; some lentiginosine synthetic derivatives [20], [21] including the enantiomer (−)-lentiginosine (compounds **2–7**, Figure 1), were also tested. Radicicol [22] was used as positive controls. This assay allowed us to verify the affinity of compounds **1–7** toward the investigated protein, giving a detailed view about their interaction with Hsp90 (Figure 2). Six out of the seven tested compounds efficiently interacted with the immobilized protein, as demonstrated by the concentration dependent responses, and by the clearly discernible exponential curves, during both the association and dissociation phases (Figure 2A). Only (-)-lentiginosine did not bind to the molecular chaperone (Figure 2B). Compounds **1–7** were also injected on immobilized bovine serum albumin (BSA) to evaluate possible unspecific bindings, and they did not interact with this protein.

Sensorgrams acquired for those compounds interacting with Hsp90 were fitted to a single-site bimolecular interaction model (A+B = AB): using this approach, kinetic and thermodynamic parameters for each complex formation were achieved (Table 1). Each constant was calculated fitting at least 12 curves, obtained by injecting three times the investigated iminosugars at four different concentrations, ranging from 0.020 µM to 1 µM. (+)-Lentiginosine (**1**) and compound **4** showed the higher affinity towards the chaperone, as inferred by the 24.7 nM and 45.9 nM *K_D_*, measured for the interaction of these compounds with Hsp90. Interestingly, compounds **1**, **3–6** showed very similar kinetic dissociation constants (*k_off_*), thus suggesting that the modification made on the (+)-lentiginosine structure did not affect the stability of the complex with Hsp90, but sensibly lowered the complex formation kinetic. On the basis of SPR data some activity-structure relationship evaluations can be attempted: in particular, the introduction of a partially hydrophobic portion resulted the most efficient change of (+)-lentiginosine structure, whereas the hydrophilic harms present in compounds **3** and **5** reduced affinity towards Hsp90. This effect was confirmed by the results achieved for compound **7**, indicating that the introduction of a primary amino group on C-7 of (+)-lentiginosine worsened both kinetic and thermodynamic of its interaction with Hsp90.

**Table 1:** Thermodynamic and kinetic constants measured by SPR for the interaction between tested compounds and immobilized HSP90.

Compound	K_D_ (nM)	*k_off_* (1/s)×10^3^
**1**	24.7±0.6	1.86±0.31
**2**	No binding	
**3**	108.3±1.1	2.18±0.23
**4**	45.9±0.5	2.05±0.42
**5**	115.6±1.6	2.55±0.35
**6**	306.8±2.9	4.12±0.81
**7**	1009.4±5.8	25.6±2.1
**Radicicol**	1.7±0.4	0.15±0.03

**Figure 3:**
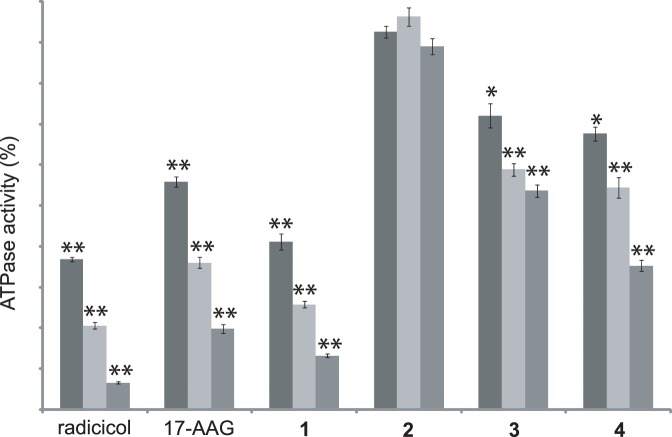
Inhibition of the ATPase activity of Hsp90 by different concentration of compounds 1–4. Radicicol and 17-AAG were used as positive controls. Data are the mean of two independent experiments performed in triplicate and were analyzed by t test (Hsp90 vs Hsp90+ testing compound): *P<0.05, **P<0.005.

To evaluate the effect of interacting compounds on Hsp90 biological activity, the ATPase activity of the enzyme was tested in the presence of the most affine compounds (**1**, **3** and **4**); as positive controls we selected radicicol and 17-AAG, two of the most potent inhibitors of the ATPase activity of the chaperone [23]. (−)-Lentiginosine was used as negative control. The obtained results (Figure 3) demonstrated that compounds **1**, **3** and **4** inhibit the ATPase activity of Hsp90 in a concentration dependent manner and the efficiency of these compounds was in agreement with their affinity towards the chaperone, as measured by SPR. Moreover, compound **1** showed an inhibitory power higher than that measured for 17-AAG.

**Figure 4:**
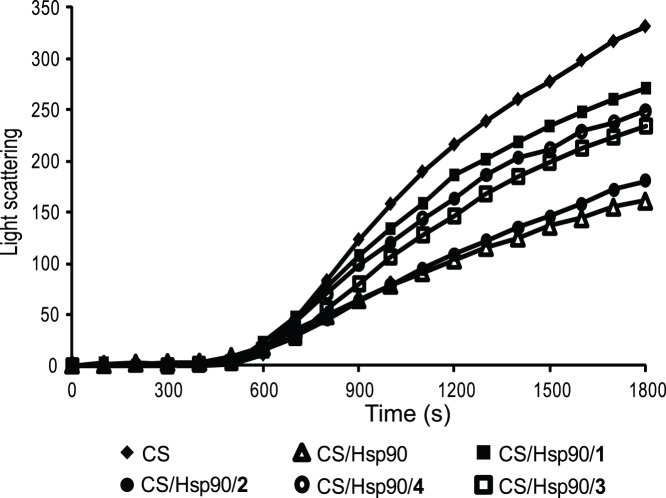
Aggregation kinetics of CS at 43°C determined by light scattering. The spontaneous aggregation of CS at 43**°**C (♦) and the aggregation of CS at 43**°**C in the presence of 0.075 µM Hsp90 (Δ) or of 0.075 µM Hsp90 and 0.3 µM compound **1** (▪), 0.3 µM compound **2** (•), 0.3 compound **3** (□), or 0.3 µM compound **4** (○) are shown.

The ability of compounds **1**, **3** and **4** to inhibit the chaperone activity of Hsp90 was also evaluated monitoring the citrate synthase (CS) thermal induced aggregation in the presence of Hsp90 [24], with or without these compounds. Again, radicicol and compound **2** were used as positive and negative controls, respectively (Figure 4). Upon incubation at elevated temperatures, CS underwent quantitative aggregation, but the presence of stoichiometric amounts of Hsp90 significantly changed the protein aggregation kinetics. When a 4-fold molar excess of **1** was added to this mixture, the curve slope increased becoming almost comparable to that observed without the chaperone, confirming the inhibition of Hsp90 chaperone activity by this compound. Similar results were achieved with **3** and **4**, even if their inhibitory effects were less evident. The addition of the same amount of **2** did not perturb the CS+Hsp90 curve, thus indicating that this compound is not able to inhibit the chaperone activity of Hsp90.

Since unmodified (+)-lentiginosine showed the higher affinity towards the chaperone and turned to be the most efficient inhibitor of its ATPase activity, we focused our attention on the mechanism of action of this compound. In an effort to identify the Hsp90 region involved in (**+**)**-**lentiginosine binding, we first performed some fluorescence polarization assays using a fluorescent geldanamycin probe (fGDA) to evaluate if this compound could interact with the ATP-binding pocket of Hsp90 [25]. In our experiments, compound **1** was unable to displace fGDA at concentration up to 100 µM, independently if it was added before or after fGDA addition; this result suggested that compound **1** acts on Hsp90 by a mechanism different from that of the currently available N-terminal inhibitors.

**Figure 5:**
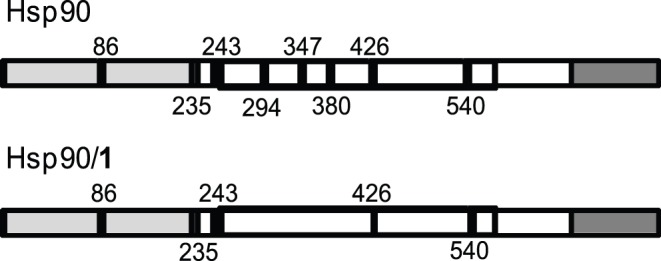
Schematic representation of the results obtained from limited proteolysis experiments. The preferential cleavage sites detected on recombinant Hsp90, and on the Hsp90/**1** complex are in black. The Hsp90 N-terminal domain is highlighted in light grey, the middle domain is boxed and the C-terminal domain is highlighted in grey.

We used limited proteolysis-mass spectrometry based strategy for the structural analysis of the Hsp90/**1** complex. This approach is based on the evidence that exposed, poorly structured, and flexible regions of a protein can be recognized by a proteolytic enzyme. The differences in the proteolytic patterns observed in presence or in absence of putative protein ligands can be analyzed to identify the protein regions involved in the molecular interactions [26]. The efficiency of this approach in investigation of Hsp90/inhibitor complexes was recently demonstrated [25]. The differences in proteolytic patterns observed on Hsp90 or on Hsp90/**1** complex (Figure 5) confirmed a direct interaction occurring between (+)-lentiginosine and the chaperone in free native state, and demonstrated that Arg279, Lys336 and Lys366, hydrolyzed in the experiments performed on the isolated protein, were protected in the complex, suggesting an interaction mainly involving the Hsp90 middle domain.

**Figure 6:**
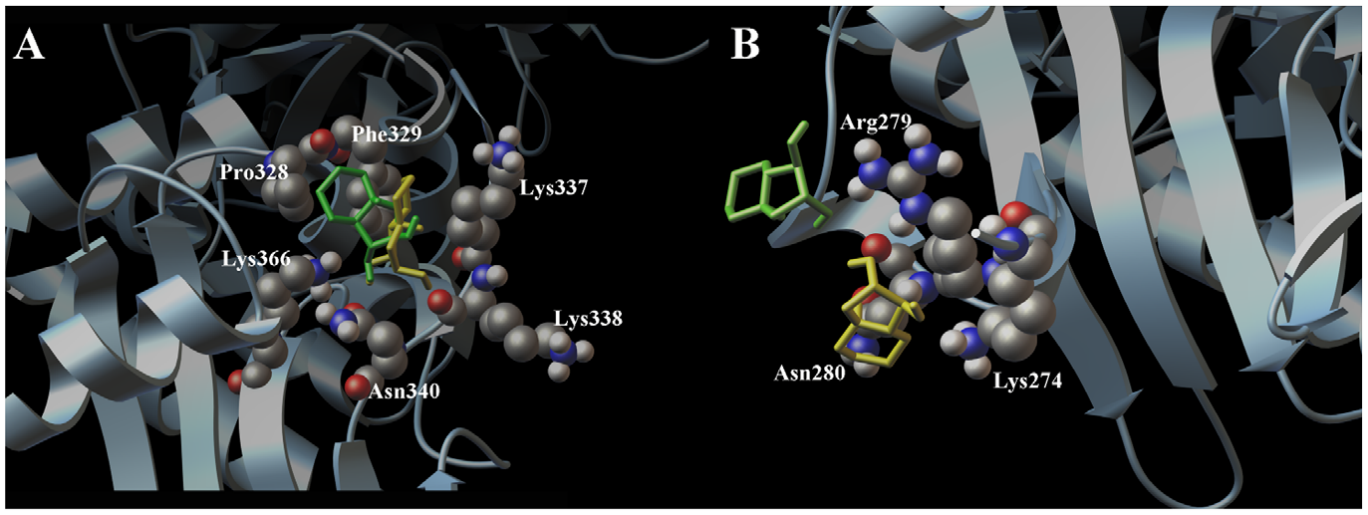
Docking calculation results. Three dimensional models (A and B) of (+)-lentiginosine (1, yellow) and (−)-lentiginosine (2, green) with HSP90. The target molecule is depicted by sky blue ribbon and the crucial amino acids by cpk (by atom type: C, purple; O, red; N, dark blue, H, white).

In order to describe at atomic level the molecular interactions between the (+)-lentiginosine (**1**) and the (−)-lentiginosine (**2**) with Hsp90, we performed docking calculations using Autodock Vina [27] and Autodock 4.2 software [28]. For our studies we referred to a crystal structure of the yeast middle and C-terminal domain determined by Ali et al. in 2006 [29], used by us [30] and other research groups [31], and its sequence alignment with the human protein reported by Lee et al. [32] for the studies of new Hsp90 inhibitors. During our studies, we considered the lentiginosine octahydro-indolizine ring protonated at physiological pH. Prior to the docking calculations, we performed a conformational search by means of molecular dynamics at different temperatures (300 and 750 K) and by Monte Carlo Method using the OPLS [33] force field included in the MacroModel software package [34]. As shown in Figure 6A, both **1** and **2** are able to interact with the middle-domain of Hsp90, establishing hydrophobic interactions and hydrogen bonds with protein side-chains (Lys337, Phe329, Pro328). The similar h-bonds in the models Hsp90/**1** and Hsp90/**2** were observed, between the OH at C-1 for both **1** and **2** with the Lys366 and Asn340, whereas their cyclic portion are located on opposite sides of the receptor surface hydrophobic pocket. On the other hand, only the OH at C-2 of **1** is able to establish a further hydrogen bond with Lys338.

Besides, our docking calculations showed a different spatial arrangement of **1** and **2** around the amino acid Arg279 (Figure 6B), identified as involved in the inter-molecular interaction by the MS experiments. In particular, even if both the molecules form a hydrogen bond with this key amino acid, **1** forms further and specific hydrogen bond with Lys274 and establishes a hydrophobic contact with Asn280. Thus, the results of docking calculations supported the view that (+)-lentiginosine preferentially interacts with the middle domain, as previously disclosed by MS analysis.

The higher number of hydrophobic and hydrogen bond interactions of **1** with respect to **2** is in agreement with SPR results, and suggest that these contacts may be critical for the different ability in the modulation of the biological activity of Hsp90 showed by these molecules. Our results indicated that (+)-lentiginosine modulates Hsp90 action by affecting its ATPase activity, even if it does not directly interact with the ATP binding site. Indeed, our limited proteolysis and docking data indicated that (**+**)**-**lentiginosine binds Hsp90 middle domain. This result is not surprising since structural studies performed on Hsp90 by different approaches demonstrated that intra-domain and inter-domain interactions cooperatively stabilize the active conformation of Hsp90 ATPase active site [35]. Specifically, Hsp90 middle domain indirectly and directly participates in the ATP hydrolysis: the co-chaperones Aha-1 binds the Hsp90 middle domain and modulated the ATPase activity of the enzyme [36]; besides, a loop involving residues 376–380 stabilize the ATP binding site, both by hydrophobic interaction and by the direct linkage between Arg380 and the γ-phosphate of bound ATP [35]. Moreover, most of the client and co-chaperones interact with the middle domain of Hsp90 [7], [8], and the binding of (**+**)**-**lentiginosine to that protein region could also mask residues playing a critical role in the recognition of substrates. From our data it can therefore be deduced that the binding of (**+**)**-**lentiginosine to Hsp90 affects some inter-domain interactions, resulting in a sensible decrease in the activity of the chaperone.

The anti-Hsp90 effects of (+)-lentiginosine, together with its anti-amyloglucosidases activity could therefore indicate this compound as a very interesting lead for the design of a new class of multifunctional inhibitors. Moreover, the selective binding to Hsp90 of (+)-lentiginosine with respect to (–)-lentiginosine suggests this compound as an useful probe to perform further conformational studies on the Hsp90 middle domain. Indeed, the middle domain of Hsp90, as opposed to its ATP-binding pocket, appears to be a promising binding site for Hsp90 client selective inhibitors (30). Further studies on the interaction of the other compounds (3–7), which showed a binding with the chaperone, will allow a better definition of the interaction surface on the Hsp90 middle domain, and to design new more active compounds.

## Materials and Methods

### General Procedures

MALDITOF/MS spectra were acquired by a MALDI micro MX (Waters); SPR analyses were performed using a Biacore 3000 optical biosensor equipped with research-grade CM5 sensor chips (GE Healthcare, Milano, Italy). Light scattering measurements were performed with a Perkin Elmer LS 55 fluorimeter (Perkin Elmer, Zaventem, Belgium) equipped by a water thermostatable cell holder with stirrer.

#### Materials

Compounds 1–7 were synthesized according to the literature [20], [21]. Solvents (HPLC grade) were purchased from Romil (ROMIL Ltd, Cambridge, UK). All buffers were prepared with a Milli-Q water system (Millipore, Bedford, MA, USA). Recombinant human Hsp90 and yeast Hsp82, provided by Assay Designs (Ann Arbor, MI, USA), were cloned from a HeLa cDNA library and expressed in *E. coli* and purified by multi-step chromatography. Proteomic grade trypsin was purchased from Sigma-Aldrich (Sigma-Aldrich Co, Milano, Italy).

#### Surface plasmon resonance analyses

SPR analyses were performed as describe elsewhere [37], [38]. Briefly, SPR analyses were performed using a Biacore 3000 optical biosensor equipped with research-grade CM5 sensor chips (GE Healthcare). Using this platform, two separate recombinant Hsp90 surfaces, a BSA surface and an unmodified reference surface were prepared for simultaneous analyses. Proteins (100 µg mL^−1^ in 10 mM sodium acetate, pH 5.0) were immobilized on individual sensor chip surfaces at a flow rate of 5 µL min^−1^ using standard amine-coupling protocols to obtain densities of 8–12 kRU. Compounds **1–7**, as well as radicicol used as positive controls, were dissolved in 100% DMSO to obtain 4 mM solutions, and diluted 1∶200 (v/v) in PBS (10 mM NaH_2_PO_4_, 150 mM NaCl, pH 7.4) to a final DMSO concentration of 0.5%. Compounds concentration series were prepared as twofold dilutions into running buffer: for each sample, the complete binding study was performed using a six-point concentration series, typically spanning 0.025–1 µM, and triplicate aliquots of each compound concentration were dispensed into single-use vials. Included in each analysis were multiple blank samples of running buffer alone. Binding experiments were performed at 25**°**C, using a flow rate of 50 µL min^−1^, with 60 s monitoring of association and 200 s monitoring of dissociation. Simple interactions were adequately fit to a single-site bimolecular interaction model (A+B = AB), yielding a single K_D_. Sensorgram elaborations were performed using the Biaevaluation software provided by GE Healthcare.

### ATP Hydrolysis Inhibition

The Discover RX ADP HunterTM Plus Assay kit was used following the manufacturer’s instructions. ATPase reactions were carried out for 60 min at 40**°**C temperature in 100 mM Tris pH 7.4, 100 µM ATP and 40 nM Hsp90 in presence of different concentrations of compounds **1–4** or radicicol. ADP generation was measured using a Perkin Elmer LS 55 fluorimeter (540 nm excitation and 620 nm emission). Fluorescence intensity values measured for Hsp90 without any testing compound was assumed as 100% of enzyme activity. The background reaction rate was measured in a reaction lacking enzyme or substrate and subtracted from the experimental rates.

#### Citrate synthase aggregation assay

Chaperone activity was evaluated as reported elsewhere by monitoring the thermal-induced aggregation of Citrate synthase (CS) in absence or in presence of a stoichiometric amount of Hsp90, and with or without a 4-fold molar excess of each testing compound. Aggregation was initiated by unfolding CS (0.075 µM) incubating the protein in 40 mM HEPES–KOH, pH 7.5 at 43**°**C. Aggregation was monitored by measuring light scattering at right angles with Perkin Elmer LS 55 fluorimeter in stirred and thermostated quartz cells. Both the emission and excitation wavelengths were set at 500 nm, and the band pass was 2 nm. Kinetics traces reported here are the averages of two measurements.

### Statistical Analysis

All the reported values represent the mean±standard deviation (SD) of at least two independent experiments performed in triplicate. Where necessary, data were statistically compared by t-test.

#### Fluorescence polarization assay

Binding of compound **1** to human full length recombinant Hsp90 was determined by a competitive binding fluorescence polarization assay using a fluorescent geldanamycin probe, as reported elsewhere [25].

#### Limited proteolysis

Limited proteolysis experiments were performed on recombinant Hsp82 at 37**°**C, PBS 0.1% DMSO, using trypsin as proteolytic agent; 30 µL of a 3 µM Hsp90 solution were used for each experiment. Binary complex Hsp90/**1** was formed by incubating the protein with a 5∶1 molar excess of the inhibitor at 37**°**C for 15 min prior to proteolytic enzyme addition. Both protein and complex were digested using a 1∶100 (w/w) enzyme to substrate ratio. The extent of the reactions was monitored on a time-course basis by sampling the incubation mixture after 5, 15, and 30 min of digestion. Samples were analyzed by MALDITOF/MS using a MALDI micro MX (Waters). Mass data were elaborated using the Masslynx software (Waters). Preferential hydrolysis sites on Hsp90 under different conditions were identified on the basis of the fragments released during the enzymatic digestions.

### Computational Details

Molecular dynamics and Monte Carlo calculations were both performed on (+)-lentiginosine (**1**) and (−)-lentiginosine (**2**) on a 4× AMD Opteron SixCore at 2.4 GHz. Molecular dynamics calculations for both **1** and **2** were performed at two different temperatures (300 and 750 K for 8 ns using 1.5 fs as time-step) using the OPLS [33] force field (MacroModel software package) [34]. During the calculations, a standard constant temperature velocity−Verlet algorithm was used to integrate the equations of motions [39]. All the obtained structures (numbering of 150, selected at regular intervals throughout the simulation) from Molecular Dynamics calculations for each isomer were minimized using the Polak-Ribier conjugate gradient algorithm (PRCG, 100000 steps, convergence threshold 0.005 kJ mol^−1^ Å^−1^), leading to the selection of the lowest energy minimum conformer for both the enantiomers. The distribution of the resulting geometries were in accordance with the results of a parallel conformational search performed with the Monte Carlo multiple minimum (MCMM) method (OPLS, 10000 steps, numbering 150, stored on a similarity and an energy criterion), where the variables used for the calculations included all the possible rotatable torsions.

We performed molecular docking experiments by Autodock 4.2 software [28] on 4× AMD Opteron SixCore at 2.4 GHz. We used a grid box size of 58×22×20 and 40×24×38 for chain A of Hsp82 (pdb code:2CGE) [29] with spacing of 0.375 Å between the grid points, and centered at −58.992 (x), 33.009 (y), 1.978 (z), and at −80.075 (x), 19.979 (y), 6.43 (z). To achieve a representative conformational space during the docking studies and for taking into account the variable number of active torsions, 10 calculations consisting of 256 runs were performed, obtaining 2560 structures for each ligand. The Lamarckian genetic algorithm (LGA) was employed for docking experiments, choosing an initial population of 600 randomly placed individuals. The maximum number of energy evaluations and of generations was set up to 5×10^6^ and to 6×10^6^ respectively. For all the docked structures, all bonds were treated as active torsional bonds except the bonds in cycles, which are considered fixed together with the receptors. Results differing by less than 3.5 Å in positional root-mean-square deviation (RMSD) were clustered together and represented by the result with the most favorable free energy of binding. Moreover, we complementarily used the Autodock Vina software [27] choosing a grid box size of 28×14×12 and of 16×10×20, with spacing of 1.000 Å between the grid points, and centered −58.717 (x), 29.973 (y) and 1.927 (z), and −80.075 (x), 19.979 (y) and 6.43 (z) covering the two possible ligand binding site on the middle domain. For the docking studies, we used an exhaustiveness of 512 with maximum energy difference of 3 kcal/mol between the best binding mode and the worst one displayed.
